# Effects of factors influencing cesarean section rates between 2008 and 2018 in Taiwan: A population-based cross-sectional study

**DOI:** 10.1097/MD.0000000000040811

**Published:** 2024-12-06

**Authors:** Wing Lam Tsui, Guang-Hong Deng, Tsung-Cheng Hsieh, Dah-Ching Ding

**Affiliations:** a Department of Obstetrics and Gynecology, Hualien Tzu Chi Hospital, Buddhist Tzu Chi Medical Foundation, Tzu Chi University, Hualien, Taiwan; b Tzu Chi University Research Center for Big Data Teaching, Research and Statistic Consultation, Tzu Chi University, Hualien, Taiwan; c Institute of Medical Sciences, Tzu Chi University, Hualien, Taiwan.

**Keywords:** cesarean section, hospital, income, maternal age, physician sex

## Abstract

Many factors can affect delivery mode decisions. Therefore, this study aimed to explore the effects of maternal age, physician’s sex, region, income, and hospital type on cesarean section (C/S) delivery rates between 2008 and 2018 in Taiwan. In this population-based cross-sectional study, data were extracted from the Taiwan National Health Insurance Research Database (2 million individuals). The logistic regression method was used to analyze the aforementioned risk factors, and data are expressed as odds ratios (ORs) and 95% confidence intervals. In total, 9826 and 9714 deliveries in 2008 and 2018, respectively, were included in the analysis. The C/S ratio increased from 16.5% (n = 1607) in 2008 to 19.7% (n = 1916) in 2018. A higher C/S risk for women aged >34 years (ORs: 2.835 and 2.225 in 2008 and 2018, respectively) than for those aged ≤34 years was noted in both years. Female physicians had a lower risk of performing C/S than male physicians in 2008 (OR: .762, 95% confidence interval: .625–.928), but this was not apparent in 2018. Higher income levels (>new Taiwan dollar 45,081) and central Taiwan were associated with a lower C/S risk in both years. Private, not-for-profit hospitals had a lower C/S risk in 2008, which was not apparent in 2018. In conclusion, this study revealed a significant increase in C/S rates over the past decade, which was influenced by multiple factors. Maternal age, physician’s sex, income status, location, and type of hospital may influence C/S rates. Analyzing these relationships can inform the development of strategies aimed at reducing future C/S rates, and targeted interventions may reduce the C/S rates.

## 1. Introduction

The global prevalence of cesarean section (C/S) births continues to increase, reaching 20% worldwide in 2022 and 31.8% in the US in 2020.^[1]^ Eastern Asian countries notably surpass this at a rate of approximately 33%. The C/S rate in Taiwan ranged from 35.3% in 2008 to 36.2% in 2018.^[2,3]^ Projections have indicated further increases in the coming decades.^[4]^ C/S, while common, presents potential complications for mothers and infants, ranging from infection and hemorrhage to delayed recovery and respiratory distress in newborns.^[5–7]^ Besides the aforementioned medical condition, physician sex, maternal age, income, hospital location, and type may influence the C/S decision.

Physician sex is a noteworthy factor influencing the choice of delivery mode.^[8]^ Studies have indicated that female physicians are less inclined to perform C/S than their male counterparts.^[8]^ This phenomenon may stem from patient preferences, with female physicians demonstrating a lower tendency to opt for C/S deliveries and exhibiting a preference for shared decision-making, potentially affecting C/S rates.^[9–11]^ Additionally, research has suggested that experience, irrespective of sex, contributes to lower C/S rates, indicating enhanced clinical judgment and decision-making skills among more seasoned practitioners.^[8]^

The age of pregnant women has been identified as a significant factor influencing the likelihood of C/S deliveries.^[12]^ Advanced maternal age is associated with an increased risk of C/S delivery.^[13]^ Several studies, including a previous meta-analysis, have reported a higher prevalence of C/S among women of advanced maternal age.^[14–16]^ The risk seems to increase, particularly in women aged 34 years and older.^[14]^ The reasons for the elevated C/S rates in older mothers are multifaceted. Advanced maternal age is often associated with an increased risk of pregnancy-related complications.^[16]^ Additionally, older mothers may experience a slower progression of labor, leading to a higher likelihood of intervention through C/S delivery.^[15]^ Older mothers potentially expressed a preference for elective C/S because of concerns about the potential complications associated with vaginal delivery (VD) in their age group.^[17]^

Traditionally, studies have indicated that women with higher socioeconomic status, often measured by income levels, may have a higher likelihood of undergoing C/S delivery.^[18,19]^ This association was linked to increased access to healthcare resources, a higher prevalence of elective C/S among wealthier individuals, and a greater tendency for medical interventions in this demographic group.^[19]^

Hospital location and type play crucial roles in determining the odds of C/S delivery.^[20]^ The healthcare infrastructure, regional differences, and hospital characteristics of childbirth practices may affect the C/S. Global trends indicate that urban areas have higher C/S rates than rural areas,^[21]^ and accessibility to healthcare facilities, medical interventions, and potentially different cultural attitudes toward childbirth may contribute to this disparity. The conventional associations between private hospitals and higher C/S rates are often attributed to financial incentives. Globally, studies have indicated higher C/S ratios have been reported in urban settings than in rural settings.^[22,23]^

Acknowledging the limitations of previous observational studies, this study aimed to comprehensively assess the factors shaping C/S delivery decisions in 2008 and 2018 in Taiwan.

## 2. Methods

### 2.1. Ethics statements

All study protocols were approved by The Research Ethics Committee at Hualien Tzu Chi Hospital (permit number: IRB111-043-C). The requirement for informed consent was waived owing to the low risk of patient safety, and the study was approved by The Research Ethics Committee at Hualien Tzu Chi Hospital. All methods were performed in accordance with relevant guidelines and regulations.

### 2.2. Study design and population

In this population-based cross-sectional study, we used the Taiwan National Health Insurance Research Database (NHIRD), which covers the medical records of 23 million Taiwanese individuals. The NHIRD is based on the National Health Insurance (NHI) program, which started in March 1995, and the database used in the study included the records of 2 million people between 2000 and 2018. The central database included Ambulatory Care Expenditures by Visit (H_NHI_OPDTE), Details of Ambulatory Care Orders (H_NHI_OPDTO), Inpatient Expenditures by Admissions (H_NHI_IPDTE), Details of Inpatient Orders (H_NHI_IPDTO), and Registry for Beneficiaries (H_NHI_ENROL).

This study included pregnant women between January 1 and December 31 of 2008 and 2018. Birth was defined according to the ORDER_CODE in H_NHI_IPDTO. Pregnant women were classified into the following categories: those who underwent VD (ORDER_CODEs: 81017C, 81018C, 81024C, 81025C, 81026C, 97931K, 97932A, 97934C, 81034C, 97004C, 97005D, 97001K, 97002A, and 97003B) and those who underwent cesarean delivery (ORDER_CODEs: 81004C, 81005C, 81028C, 81029C, 97014C, 81005B, 81011C, 97006K, 97007A, 97008B, and 97009C).

Women for whom age data were not precise were excluded. Physicians of no apparent sex were excluded. This study used the International Classification of Diseases, Ninth Revision (ICD9) for the 2000 to 2015 database and the International Classification of Disease, Tenth Revision (ICD10) for the 2016 to 2018 database to diagnose diseases. Patients with pregnancy or delivery complications, such as miscarriage, stillbirth, multiple pregnancies, placenta previa, premature birth, placental abruption, dystocia, and fetal distress, were excluded from our study to maintain the focus on low-risk pregnancies and provide a consistent baseline for evaluating outcomes without confounding factors associated with high-risk cases. Including these complications would introduce substantial variability, making it difficult to achieve comparability within the study and potentially obscuring the findings related to the condition being investigated. Additionally, complicated pregnancies often require specialized care, posing ethical and safety concerns that could complicate study protocols. Excluding such cases ensures that the data more accurately reflect the target population, reduces potential biases, and supports data integrity by eliminating factors that could skew the results or limit the applicability of the findings to uncomplicated pregnancies. Additionally, women who had a miscarriage (ICD9 codes: 634–637 and 779.6; ICD10codes: O03, O04, O02.1, and Z33.2), stillbirth (ICD9 codes: 656.4, 768.0, and 768.1; ICD10 codes: P84 and O36.4XX), multiple pregnancies (ICD9 code: 651; ICD10 codes: O30 and O31.1–O31.3), placenta previa (ICD9 codes: 641.0 and 641.1; ICD10 code: O44), premature birth (ICD9 code: 644; ICD10 code: O60), placental abruption (ICD9 code: 641.2; ICD10 code: O45), breech birth (ICD9 codes: 652.2 and 669.6; ICD10 codes: O32.1 and O64.1), dystocia (ICD9 codes: 653, 660, 661.1, 661.2, 661.5–661.9, and 662; ICD10 codes: O33, O64.0, O64.9, O62.1, O62.2, O62.4–O62.9, and O63), and fetal distress (ICD9 code: 656.3; ICD10 code: O68) were excluded. These variables were excluded because they presented contraindications for either C/S or VD. Lastly, women with duplicate births in 2008 and 2018 were excluded.

### 2.3. Study classifications

This study included nonclinical factors such as patient demographics (age, birth year, income-related amount insured, and geographic region of birth), physician sex, and hospital ownership. The patients were categorized into ≤34- and >34-year-old age groups. We also divided the participants into 2 age groups based on the criteria used in Taiwan, where the >34-year-old age group is classified as “advanced maternal age.” The income-related amounts insured were categorized as ≤new Taiwan dollars (NTD) 28,800, 28,801–45,800, and ≥45,801. Geographical regions of birth were classified as northern, central, southern, eastern, or outlying islands. Hospital ownership was classified as public, private not-for-profit, and private for-profit.

### 2.4. Statistical analysis

The number and percentage of patient demographics (age, income-related amount insured, and geographic region of birth), physician sex, and hospital ownership were recorded. Logistic regression analysis was used to assess the factors influencing the likelihood of C/S delivery, including physician sex, patient age, income, geographic region of birth, and hospital ownership, in 2008 and 2018. Statistical software SAS (version 9.4; SAS Institute, Cary, NC) was used to perform data analysis, and a *P*-value < .05 was considered significant.

## 3. Results

### 3.1. Study design

**Figure 1.**
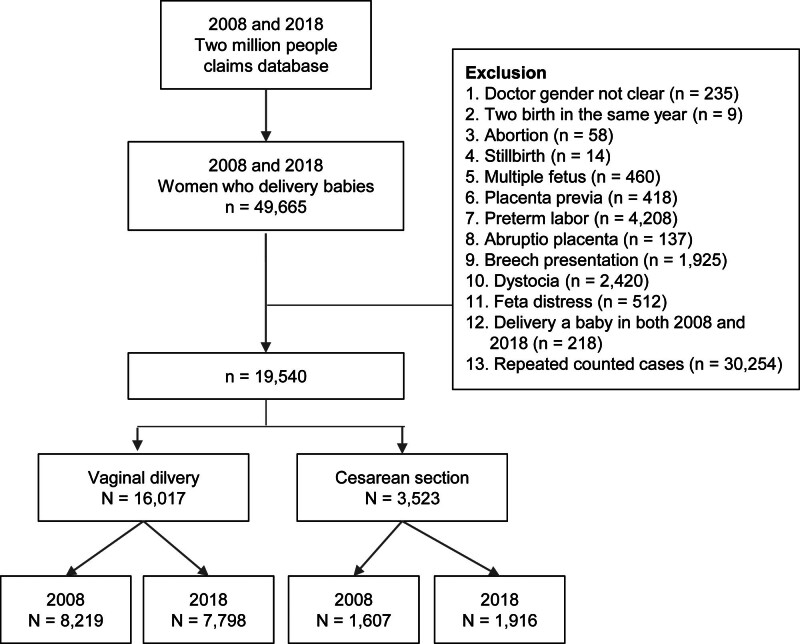
Flow chart of the study population.

Between 2008 and 2018, 49,665 women had deliveries in 2008 and 2018 (Fig. 1). After exclusion, 16,017 and 3523 deliveries were performed using VD and C/S, respectively. Finally, 8219 and 7798 deliveries occurred via the vagina in 2008 and 2018, respectively, and 1607 and 1916 deliveries occurred via C/S in 2008 and 2018, respectively.

### 3.2. Basic characteristics related to C/S in 2008 and 2018

The C/S rate increased from 16.5% in 2008 to 19.7% in 2018 (Table 1). In total, 3079 C/S deliveries were performed by male physicians (18.1%), and 444 C/S were performed by female physicians (17.8%) (Table 1). The C/S rates in women aged ≤34 and >34 years were 14.8% and 30.2%, respectively, and the C/S rates were 16.4% and 19.7% in 2008 and 2018, respectively. Northern hospitals occupied 20% of the C/S rate, which was more than other regional hospitals in Taiwan. Income level was not associated with the C/S rate (range, 17.5–18.5%). Public hospitals performed more C/S deliveries (21.1%) than other types of hospitals.

**Table 1:** Cesarean deliveries in 2008 and 2018 in Taiwan by birth year, physician sex, and control variable.

Variable	Delivery mode, n (%)	
	Vaginal delivery	Cesarean delivery
Birth year		
2008	8219 (83.6)	1607 (16.4)
2018	7798 (80.3)	1916 (19.7)
Physician sex		
Male	13,967 (81.9)	3079 (18.1)
Female	2050 (82.2)	444 (17.8)
Patient age (years)		
≤34	13,184 (85.2)	2297 (14.8)
>34	2833 (69.8)	1226 (30.2)
Income-related insured amount		
≤NTD 28,800	4441 (81.5)	1007 (18.5)
NTD 28,801–45,800	5825 (82.5)	1235 (17.5)
≥NTD 45,801	5751 (81.8)	1281 (18.2)
Geographic region		
Northern	7594 (80.0)	1902 (20.0)
Central	4064 (84.9)	723 (15.1)
Southern	3963 (83.3)	820 (17.1)
Eastern	339 (85.1)	68 (16.7)
Outlying islands	57 (82.0)	10 (14.9)
Hospital ownership		
Public	1459 (78.9)	390 (21.1)
Private not-for-profit	5716 (82.9)	1175 (17.1)
Private for-profit	8842 (81.9)	1958 (18.1)

NTD = new Taiwan dollar.

### 3.3. Factors influencing C/S likelihood in 2008

Table 2 presents the factors influencing the likelihood of C/S in 2008. Female physician (odds ratio [OR] = .762, 95% confidence interval [CI]: .625–.928), income level > NTD 28,800 (OR = .803–.851), hospitals located in central Taiwan (OR = .798, 95% CI = .691–.921), and private nonprofit hospitals (OR = .808, 95% CI = .665–.981) had lower odds of C/S than their counterparts. Women aged >34 years were associated with a higher risk of C/S than those aged ≤34 years (OR = 2.835, 95% CI: 2.481–3.239).

**Table 2:** Results of logistic regression analysis of the cesarean section likelihood in 2008.

Variable	Univariate analysis		Multivariate analysis†	
	OR, 95% CI	*P*-value	OR, 95% CI	*P*-value
Physician sex				
Male (reference group)	1.000		1.000	
Female	.793 (.653–.963)	.019*	.762 (.625–.928)	.007*
Patient age (years)				
≤34 (reference group)	1.000		1.000	
>34	2.842 (2.492–3.241)	<.001*	2.835 (2.481–3.239)	<.001*
Income-related insured amount				
≤NTD 28,800 (reference group)	1.000		1.000	
NTD 28,801–45,800	.864 (.756–.988)	.033*	.851 (.742–.976)	.021*
≥NTD 45,801	.907 (.796–1.034)	.145	.803 (.700–.922)	.002*
Geographic region				
Northern (reference group)	1.000		1.000	
Central	.806 (.704–.924)	.002*	.798 (.691–.921)	.002*
Southern	.906 (.794–1.033)	.141	.907 (.791–1.041)	.165
Eastern	.938 (.648–1.358)	.734	.940 (.644–1.372)	.748
Outlying islands	1.186 (.395–3.556)	.761	1.003 (.325–3.100)	.995
Hospital ownership				
Public (reference group)	1.000		1.000	
Private not-for-profit	.772 (.640–.932)	.007*	.808 (.665–.981)	.031*
Private for-profit	.747 (.624–.895)	.002*	.838 (.694–1.011)	.066

CI = confidence interval, NTD = new Taiwan dollar, OR = odds ratio.

† Adjusted for physician sex, patient age, income-related insurance amount, geographic region, and hospital ownership.

* *P* < .05.

### 3.4. Factors influencing C/S likelihood in 2018

Table 3 presents the factors influencing the likelihood of C/S in 2018. Physician sex and hospital type were not associated with the C/S rate. Income level ≥NTD 45,801 (OR = .844, 95% CI = .737–.966), hospitals located in central Taiwan (PR = .641 95% CI = .560–.733), and hospital located in south Taiwan (OR = .760, 95% CI = .668–.866) had a lower odds of C/S than their counterparts. Similar to 2008, women aged >34 years were associated with a higher risk of C/S than those aged ≤35 years (OR = 2.225, 95% CI = 2.001–2.475).

**Table 3:** Results of logistic regression analysis of cesarean section likelihood in 2018.

Variable	Univariate analysis		Multivariate analysis†	
	OR, 95% CI	*P*-value	OR, 95% CI	*P*-value
Physician sex				
Male (reference group)	1.000		1.000	
Female	1.046 (.913–1.197)	.518	1.032 (.899–1.185)	.654
Patient age (years)				
≤34 (reference group)	1.000		1.000	
>34	2.222 (2.002–2.466)	<.001*	2.225 (2.001–2.475)	<.001*
Income-related insured amount				
≤NTD 28,800 (reference group)	1.000		1.000	
NTD 28,801–45,800	.982 (.864–1.115)	.774	.895 (.785–1.019)	.095
≥NTD 45,801	1.051 (.925–1.195)	.447	.844 (.737–.966)	.014*
Geographic region				
Northern (reference group)	1.000		1.000	
Central	.635 (.557–.722)	<.001*	.641 (.560–.733)	<.001*
Southern	.764 (.674–.866)	<.001*	.760 (.668–.866)	<.001*
Eastern	.695 (.475–1.017)	.061	.832 (.562–1.231)	.357
Outlying islands	.499 (.211–1.180)	.113	.483 (.200–1.165)	.105
Hospital ownership				
Public (reference group)	1.000		1.000	
Private not-for-profit	.771 (.647–.919)	.004*	.845 (.704–1.014)	.070
Private for-profit	.914 (.774–1.079)	.288	1.066 (.896–1.269)	.469

CI: confidence interval, NTD = new Taiwan dollar, OR = odds ratio.

† Adjusted for physician sex, patient age, income-related insurance amount, geographic region, and hospital ownership.

* *P* < .05.

## 4. Discussion

Our cross-sectional study, using an NHIRD of 2 million people, enrolled 49,665 deliveries in 2008 and 2018, which were analyzed, with 16,017 and 3523 deliveries by the vaginal and C/S methods, respectively. The C/S rate increased from 16.5% in 2008 to 19.7% in 2018. A higher C/S risk for women aged >34 (ORs: 2.835 and 2.225 in 2008 and 2018, respectively) compared with those aged ≤34 years was noted in both years. Female physicians had a lower ratio of performing C/S than male physicians in 2008, but this was not apparent in 2018. Higher income levels (>NTD 45,081) and hospitals located in central Taiwan had a lower C/S risk in both years. Private, not-for-profit hospitals had a lower C/S risk in 2008, which was not apparent in 2018.

Maternal age may have influenced the odds of C/S. Our study found that the OR of advanced maternal age for C/S was the same between the 2 years. A previous study revealed an increased C/S rate in primiparas aged >34 years compared with those aged <25 or 25 to 34 years.^[14]^ Additionally, a population study showed an increased C/S rate in women of advanced maternal age.^[15]^ In a Japanese study, the risk of C/S increased with advanced maternal age.^[24]^ Although private and public institutions covered insurance, advanced maternal age contributed to higher C/S rates.^[25]^ A hospital-based study also revealed that advanced maternal age was associated with a higher C/S rate.^[26]^ Our study agrees with these studies in that advanced age may be associated with an increased likelihood of C/S. The causes of the association between advanced maternal age and increased C/S rate may include increased complication rates, decreased uterine tone, increased rates of induction, higher rates of fetal distress, and maternal health conditions^[27]^

The increased rate of C/S deliveries performed by female physicians in 2018 compared with that in 2008 could be influenced by various factors. First, medical guidelines and practices may have evolved, leading to a higher inclination toward C/S in certain cases.^[28]^ Second, women today have different preferences and expectations regarding childbirth than they had a decade ago.^[29]^ Third, physicians may be more cautious about complications during childbirth because of increased legal scrutiny and malpractice claims.^[30]^ Fourth, similar to male physicians, female physicians may face increased workloads and time constraints in modern healthcare settings.^[31]^ Lastly, financial considerations, such as reimbursement rates for C/S versus VD, could also influence physicians’ decision-making processes.^[32]^ Nevertheless, the reimbursement rates for C/S and VD were the same in Taiwan.

Globally, the C/S trend persists, with the United States reporting a rate of approximately 31.8% in 2020.^[33]^ Eastern Asian countries, however, documented the highest C/S rate of approximately 33%.^[1]^ Experts anticipate a further increase in this trend over the next decade.^[4]^ Our study also showed that C/S rates increased in 2018 compared with those in 2008.

A previous study found that the primary C/S was directly associated with socioeconomic status, and the latest research revealed that high-income women had a higher C/S rate.^[18,19]^ Another previous study revealed high C/S rates in high-income countries.^[34]^ Conversely, we found that higher income levels were associated with fewer C/S deliveries between 2008 and 2018. The causes of the correlation between high income and low C/S rates may include better access to quality prenatal care, healthier lifestyles, lower prevalence of risk factors, higher education levels and health literacy, greater autonomy in birth choice, and reduced financial incentives for medical interventions.^[35]^

A previous study showed a higher C/S rate in the most advantaged groups than in poor and rural groups.^[36]^ Multivariate research in Vietnam has also revealed a significantly higher C/S rate in urban areas than in rural areas.^[23]^ This trend has also been observed globally. Betran et al observed higher C/S rates in Eastern Asia, Western Asia, and Northern Africa than in sub-Saharan Africa and North America.^[1]^ Additionally, a study in Nepal revealed a higher C/S rate in urban areas than in rural areas, possibly because of the higher adverse outcomes of C/S in rural settings.^[37]^ In 2008 and 2018, women in northern Taiwan (more urban areas) had higher odds of C/S, which is comparable with previous studies’ findings.

A prior study revealed that the C/S rate in women with private insurance was higher than that in women with public insurance.^[25]^ An earlier study demonstrated a higher prevalence of C/S among women covered by private insurance than among those covered by public insurance.^[25]^ The latest meta-analysis showed that the C/S rate was higher in profit hospitals than in nonprofit hospitals.^[38]^ Further, a study conducted in Americans revealed that the C/S rate was highly associated with the type of insurance plan.^[39]^ In Taiwan, the fee for C/S delivery is the same as that for VD. Therefore, no motivation for selecting a particular mode of childbirth was observed. However, in our study, women in private, not-for-profit hospitals had lower odds of C/S than those in public hospitals in both years. The reasons why the C/S rate was high in public hospitals may include different patient populations, provider practices and cultures, financial incentives, resource availability and workload, patient preferences, and informed decision-making.^[40]^

Our study provides several novel aspects. First, there was a temporal trend in Taiwan’s C/S rate (from 2008–2018). This longitudinal perspective allows the identification of changes in C/S practices over time, which may reflect shifts in clinical guidelines, healthcare policies, or societal attitudes toward childbirth. Second, using the NHIRD to examine a cohort of 2 million individuals enhanced the robustness and generalizability of the findings. This comparative analysis elucidates the dynamic nature of childbirth practices and the associated risk factors, offering valuable information for guiding future interventions and healthcare policies to reduce unnecessary C/S rates. We identified several sociodemographic and healthcare provider factors related to C/S delivery, including maternal age, physician sex, income level, geographical region, and hospital type. This comprehensive examination of multiple factors provides a nuanced understanding of the complex determinants influencing C/S rates and highlights potential areas for targeted interventions and quality improvement initiatives within the healthcare system.

This was a cross-sectional study with a large sample size. The strength of a cross-sectional study lies in its ability to provide a snapshot of a population at a single point in time, allowing the assessment of the prevalence and exploration of associations between variables. Other strengths include its wide applicability, association identification, baseline information, and useful for planning.

However, our study has some limitations. First, we did not consider other qualities of obstetricians, such as age, ethnicity, background, and experience, which may also affect their delivery decision. Cultural beliefs and customs can also influence the C/S rate. Nevertheless, the available databases require specific information for analysis. However, as this was a retrospective study, we could not control for other confounding factors. Moreover, female physicians were not the only factors affecting the C/S rate.

## 5. Conclusions

This study observed a significant increase in C/S rates over the decade, with younger maternal age, higher income levels, and hospitals located in central Taiwan correlating with lower risks of C/S delivery. Sex disparity among physicians performing C/S deliveries decreased over time, and the association between private, not-for-profit hospitals and lower C/S risk diminished by 2018. These insights empower pregnant women to participate in shared decision-making with their healthcare providers, facilitating more informed and personalized healthcare choices. Analyzing these relationships can inform the development of strategies aimed at reducing future C/S rates, and targeted interventions may reduce the C/S rates.

## Acknowledgments

The authors thank the Tzu Chi University Research Center for Big Data Teaching, Research, and Statistics Consultation for providing statistical consultation assistance.

## Author contributions

**Conceptualization:** Dah-Ching Ding.

**Data curation:** Guang-Hong Deng.

**Formal analysis:** Guang-Hong Deng.

**Funding acquisition:** Dah-Ching Ding.

**Investigation:** Guang-Hong Deng.

**Project administration:** Dah-Ching Ding.

**Supervision:** Dah-Ching Ding.

**Writing – original draft:** Wing Lam Tsui, Guang-Hong Deng, Dah-Ching Ding.

**Writing – review & editing:** Wing Lam Tsui, Guang-Hong Deng, Tsung-Cheng Hsieh, Dah-Ching Ding.
